# Cell tropism of adeno-associated viruses within the mouse inner ear in vivo: from embryonic to adult stages

**DOI:** 10.1038/s41598-025-98007-x

**Published:** 2025-04-18

**Authors:** Sepideh Iranfar, Maxence Cornille, Mauricio Saenz Roldan, Baptiste Plion, Marie-José Lecomte, Saaid Safieddine, Ghizlene Lahlou

**Affiliations:** 1 Université Paris Cité, Institut Pasteur, AP-HP, INSERM, CNRS, Fondation Pour l’Audition, Institut de l’Audition, IHU reConnect, 63 Rue de Charenton, Paris, 75012 France; 2 Ecole Doctorale Physiologie, Physiopathologie et Thérapeutique, Sorbonne Université, Paris, France; 3 Centre National de la Recherche Scientifique, Paris, 75016 France; 4 Département d’Oto-Rhino-Laryngologie et de Chirurgie Cervico-Faciale, GHU Pitié-Salpêtrière, APHP Sorbonne Université, Paris, 75013 France; 5 Centre de Références Maladies Rares «Surdités génétiques», GHU Pitié-Salpêtrière, APHP, Sorbonne Université, Paris, 75013 France

**Keywords:** Inner ear, Gene therapy, AAV, Serotype, Tropism, Therapeutic time window, Molecular medicine, Preclinical research, Translational research, Cochlea, Hair cell, Inner ear, Transduction, Molecular neuroscience

## Abstract

Adeno-associated virus (AAV)-based gene therapy is emerging as a promising treatment for deafness and vestibular deficits, due to the variety of available serotypes that offer a large range of cell targeting capabilities. Nevertheless, the tropism of these AAV serotypes for sensory inner ear cells varies greatly as the cochlea matures, presenting a significant burden for successful preclinical trials. Therefore, identifying serotypes with strong tropism for cochlear and vestibular hair cells during key stages of development in mouse inner ear, the most widely used preclinical model, is essential for advancing clinical applications. We conducted a comparative analysis of the cellular tropism and hair-cell transduction rates of four AAV serotypes in the cochlea and vestibular organs during maturation. We used AAV2, AAV8, AAV9-PHP.eB, and Anc80L65 at the embryonic, neonatal, and adult stages. Our results indicate that the cell transduction rate of these four serotypes varies with age. Notably outer hair cells were mostly targeted during the embryonic stage, inner hair cells were primarily transduced principally at the mature stage, and vestibular hair cells were the most permissive at the neonatal stage. These results provide new insights for preclinical gene therapy studies for the inner ear with potential implications for therapeutic outcomes.

## Introduction

Deafness is the leading inherited sensory disorder in humans and presents a major challenge to public health systems globally. Hearing loss has a considerable impact, affecting about 430 million people worldwide who struggle with its consequences^1^, including communication difficulties, isolation, and dependence^2^. Almost one in every 700 children is affected by congenital deafness and approximately 70% of these cases are due to genetic factors^2^. Depending on the specific deafness gene involved, hearing loss may be associated with a vestibular deficit, as observed in about 30% of children with congenital deafness^3^. Among non-syndromic genetic deafness, most pathogenic variants cause defects to sensory hair cells in the inner ear^4,5^. There is currently no curative treatment for hearing or vestibular deficits. Hearing aids and cochlear implants are used for hearing rehabilitation but do not fully restore normal hearing^6,7^. For patients with balance problems, whether isolated or associated with deafness, symptomatic treatment and vestibular rehabilitation are the only available interventions^8^.

AAV-mediated gene therapy has recently emerged as a potentially curative treatment for hereditary deafness with or without associated balance disorders^9^. The first clinical trials of AAV-mediated gene therapy for DFNB9 human deafness, an autosomal recessive congenital form of deafness caused by pathogenic *OTOF* variants^10^, were recently conducted and yielded promising results supporting broader development of this approach^11,12^. Recombinant associated adenoviruses (AAVs) are currently the vector of choice for inner ear gene therapy due to their various serotypes, which enable broad cell tropism, as well as their low immunogenicity, and capacity to transduce non-dividing cells such as inner ear sensory cells^13^. The inner ear houses both the vestibule, the balance organ, and the cochlea, the organ of hearing. Both organs harbor sensory hair cells responsible for transmitting signals to the auditory and vestibular nerve pathways. These include the inner and outer hair cells (IHCs and OHCs) in the cochlea, and the vestibular hair cells (VHCs) in the vestibule^2^. They are surrounded by supporting cells that help maintain the homeostasis of the sensory epithelium.

In mice, the most commonly used model for preclinical investigations, AAV serotypes show highly variable levels of efficiency and specificity in targeting inner ear hair cells, depending on the developmental stage of cochlea at the time of administration.

It is therefore crucial to pinpoint the exact stage of inner ear development at which administrating an AAV serotype carrying therapeutic transgenes results in highest rates of specific transduction in the target cells. This step is paramount to advance basic research and to translate gene therapy into clinical applications for treating deafness and vestibular disorders. Here, we aimed to investigate and compare the cell tropism and transduction efficiency of several AAVs in cochlear and vestibular hair cells at three key developmental stages in mice: at the embryonic stage (just after hair-cell differentiation), at perineonatal stages (during inner-ear maturation before hearing onset), and at the mature stage (after hearing onset). We studied four AAV serotypes: two naturally occurring (AAV2 and AAV8) and two engineered variants (Anc80L65^14^ and AAV9-PHP.eB^15^). All the serotypes expressed the green fluorescent protein (GFP) gene under the control of ubiquitous promoters and were injected into the inner ear in wild-type mice at the developmental stages specified.

## Results

### Cell tropism of AAV vectors in the embryonic inner ear

Injections were performed between days 13 and 15 of embryonic development (E13 to E15). The viral preparation (1 µL) was injected directly into one of the otocysts of each embryo in the litter. The cochlear and vestibular sensory epithelia of each pup were microdissected on postnatal day 8 (P8) to obtain whole-mount preparations, which were immunostained for GFP and the hair-cell marker Myosin7A. The samples were then analyzed using confocal imaging, which revealed GFP expression in the cochlear and vestibular sensory cells, but with a marked variation in cellular tropism and transduction rate.

**Fig. 1:**
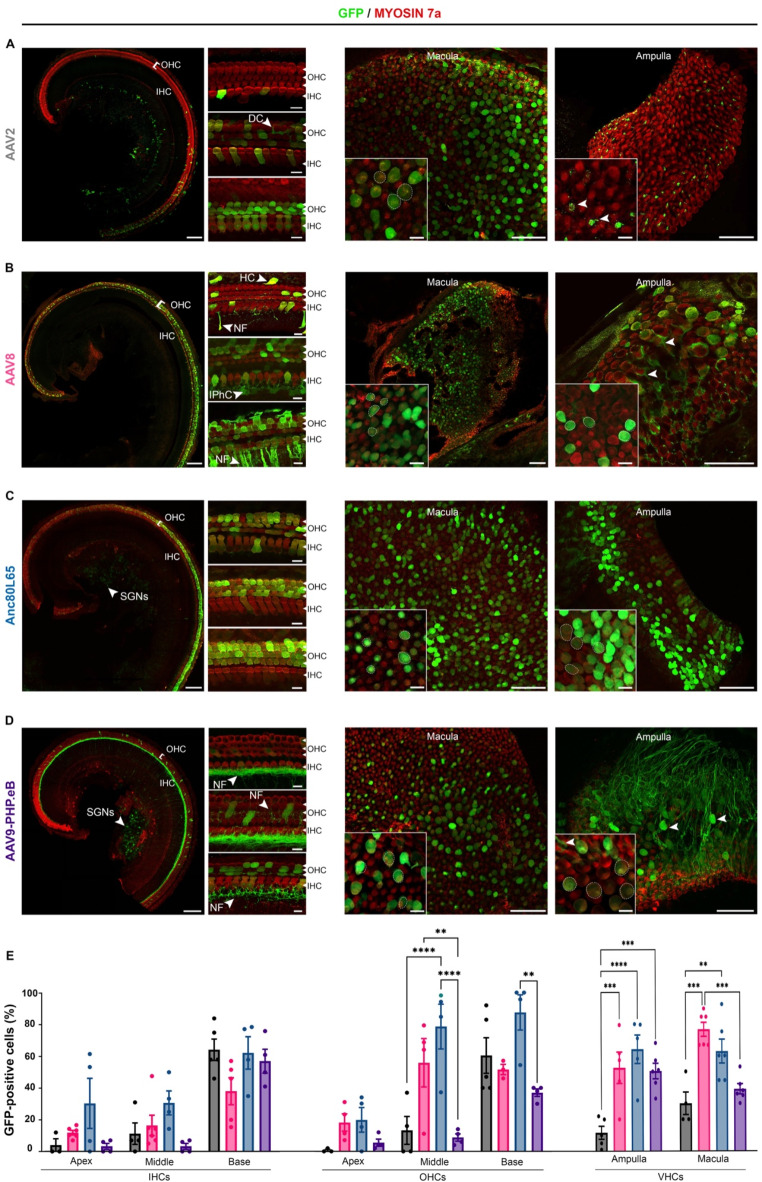
Comparison of the inner ear cell tropism of four AAV serotypes at the embryonic stage. Confocal images of the apical turn of the organ of Corti (left panels) and vestibular organs (right panels) after the injection into the otocyst on E13-E15 of 1 µL of (**A**) AAV2-CBA-GFP, (**B**) AAV8-CMV-GFP, (**C**) AAV-Anc80L65-CMV-GFP or (**D**) AAV9-PHP.eB-CBA-GFP and immunostaining on P8 for GFP (green) and myosin 7a (red). For the cochlea, the insets are close-up views of the apical (top), medial (middle), and basal (bottom) regions (scale bars: A-D, 100 μm; insets, 5 μm). IHCs, inner hair cells; OHCs, outer hair cells; SGNs, spiral ganglion neurons; DC, Deiters’ cells; NF, nerve fibers; IPhC, inner phalangeal cells, and HC, Hensen’s cells. The right panel shows a low magnification of the macula and ampulla cristae with close-up views shown in the insets (scale bars A-D, 50 μm; insets, 5 μm). GFP-positive vestibular hair cells are outlined by a dashed line and cells other than the sensory hair cells are indicated by arrowheads. (**E**) *Bar graphs* showing the percentage of hair cells—IHCs, OHCs and VHCs—transduced with AAV2 (black), AAV8 (pink), Anc80L65 (blue), and AAV9-PHP.eB (purple), (mean percentage ± SEM). Two-way ANOVA, **P < 0.01, ***P < 0.001 and ****P < 0.0001.

Quantitative analyses demonstrated substantial variation in the transduction rates of both IHCs and OHCs across the four serotypes (Fig. 1). However, the profile of transduction efficiency in all cases followed a baso-apical gradient, with greater number of cells transduced in the basal region than at the apex, (*p* < 0.0001 for comparisons of IHC transduction rates between the base and the apex and between the base and the medial turn, two-way ANOVA; *p* = 0.0004, *p* < 0.0001 and *p* = 0.01 for comparisons of OHC transduction rates between the apex and the medial turn, the apex and the base, and the base and the medial turn, respectively; Fig. 1; Table 1).

Cochlear sensory cell transduction rates were the highest with Anc80L65, which infected both IHCs and OHCs along the entire cochlear spiral, with mean transduction rates of 41 ± 8.2% and 62 ± 7.7%, respectively. In contrast, AAV9-PHP.eB showed the lowest transduction rate, with 21 ± 1.9% for IHCs and 17 ± 1.4% for OHCs (Fig. 1; Table 1). In addition, transduction rates were higher for OHCs than for IHCs for both AAV8 (44 ± 2.5% and 24 ± 4.6, respectively, *p* = 0.02, two-way ANOVA) and Anc80L65 (62 ± 7.7% and 41 ± 8.2%, respectively, *p* = 0.04, two-way ANOVA).

We also found that both supporting cells and spiral ganglion neurons (SGNs) were transduced. Precisely, supporting cells, such as inner pillar cells, inner phalangeal cells, and Deiters cells have been slightly targeted by AAV8 and AAV2. Surprisingly, at the embryonic stage, AAV9-PHP.eB transduced very few hair cells but widely infected the SGNs (Fig. 1).

For the vestibular organs, transduction rates ranged from 45 to 66% of VHCs for AAV9-PHP.eB, AAV8, and Anc80L65, with no significant difference between these three serotypes. However, transduction rates were lower for the AAV2 serotype (20 ± 4.8%; *p* < 0.0001, one-way ANOVA, Table 1; Fig. 1). VHC transduction rates were similar between the otolithic macula and cristae ampullae for all serotypes tested (Table 1, *ns*, two-way ANOVA). Except for Anc80L65, the serotypes tested displayed targeting of non-sensory vestibular cells. Notably, the vestibular nerve fibers displayed robust GFP immunostaining indicating a high transduction rate of primary vestibular neurons following embryonic injection of AAV9-PHP.eB (Fig. 1D).

**Table 1:** Transduction rates of inner ear hair cells after the injection of various AAV serotypes at the embryonic stage.

	AAV2	AAV8	Anc80L65	AAV9-PHP.eB	*p*
IHC	*n* = 5	*n* = 6	*n* = 4	*n* = 4	
Global	29 ± 1.6 [25–32]	24 ± 4.6 [11–43]	41 ± 8.2 [25–63]	21 ± 1.9 [16–25]	0.064
Apex	4 ± 4 [0–12]	12 ± 1.4 [7–16]	30 ± 15.8 [0–53]	3 ± 1.9 [0–7]	0.099
Medial turn	11 ± 6.8 [0–31]	20 ± 10.2 [5–70]	31 ± 7.5 [14–50]	3 ± 1.9 [0–7]	**0.018**
Base	64 ± 6.8 [46–84]	38 ± 8.5 [15–57]	62 ± 10.2 [35–79]	57 ± 7.5 [41–76]	0.16
OHC	*n* = 5	*n* = 6	*n* = 4	*n* = 4	
Global	31 ± 7.1 [16–50]	44 ± 2.5 [38–50]	62 ± 7.7 [41–76]	17 ± 1.4 [15–21]	**0.002**
Apex	1 ± 0.7 [0–2]	11 ± 1.3 [7–14]	20 ± 7.7 [0–34]	6 ± 2.2 [2–12]	**0.046**
Medial turn	13 ± 8.8 [0–37]	56 ± 15.3 [11–79]	79 ± 14.1 [37–98]	9 ± 2.3 [4–14]	**0.004**
Base	60 ± 11.2 [40–92]	52 ± 3.2 [46–57]	88 ± 11.1 [54–100]	37 ± 2.4 [30–40]	**0.003**
VHC	*n* = 5	*n* = 6	*n* = 4	*n* = 4	
Global	20 ± 4.8 [1–50]	66 ± 6.1 [20–90]	63 ± 8.5.5 [32–92]	45 ± 3.3 [30–68]	**< 0.0001**
Ampulla	11 ± 4.1 [1–22]	52 ± 9.8 [20–80]	64 ± 8.9 [32–80]	50 ± 4.9 [33–68]	**0.0025**
Macula	30 ± 7 [20–50]	76 ± 4.4 [67–90]	63 ± 7.5 [45–92]	39 ± 3.2 [30–53]	**0.0004**

The data shown are the mean ± SEM [range]. Statistical comparisons were performed by two-way ANOVA. *N* is the number of organs analyzed. IHC = inner hair cells; OHC = outer hair cells; VHC = vestibular hair cells.

### Cell tropism of AAV vectors in the neonatal inner ear

AAVs were delivered to the neonatal inner ear via the round window membrane (RWM) on postnatal days P2 to P4. Five days after injection, the inner ears were microdissected and subjected to immunolabeling, as described above.

**Fig. 2:**
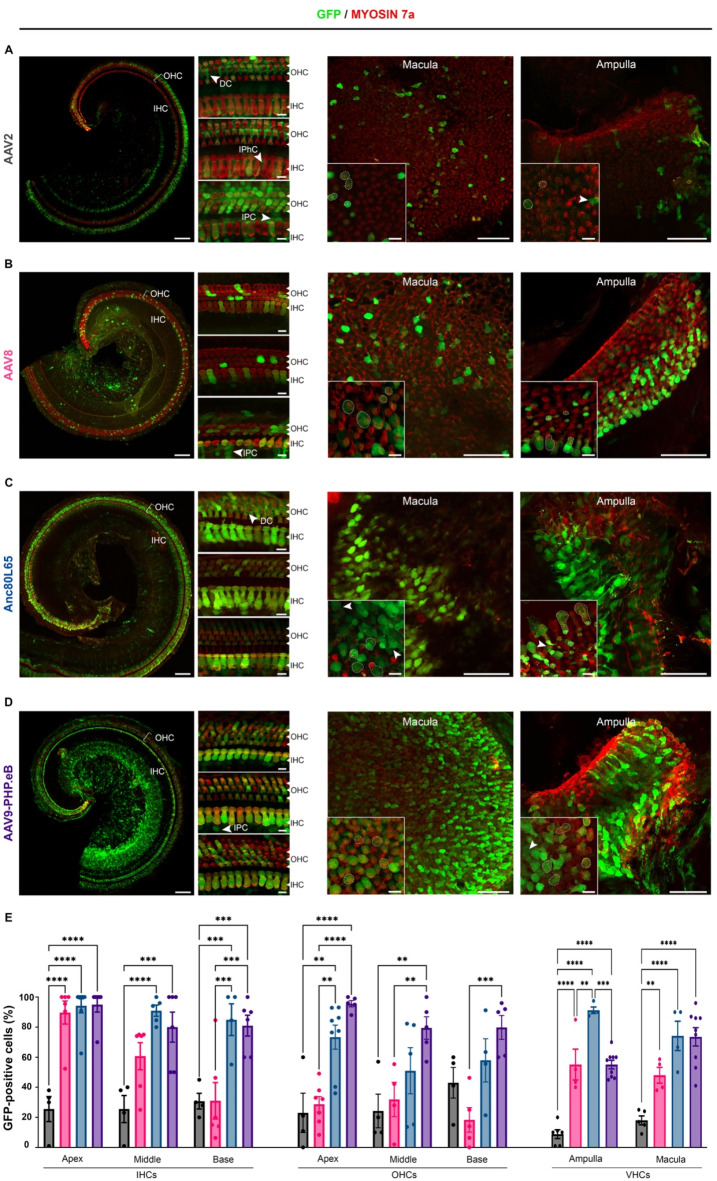
Comparison of the inner ear cell tropism of four AAV serotypes at the neonatal stage. Confocal images of the apical turn of the organ of Corti (left panels) and vestibular organs (right panels) after the injection on P2, via the RWM, of 2 µL of (**A**) AAV2-CBA-GFP, (**B**) AAV8-CMV-GFP, (**C**) AAV-Anc80L65-CMV-GFP or (**D**) AAV9-PHP.eB-CBA-GFP, followed by immunostaining on P8 for GFP (green) and myosin 7a (red). For the cochlea (left), insets are high-magnification views of the apical (top), medial (middle), and basal (bottom) regions (scale bars: **A**–**D**, 100 μm; insets, 5 μm). IHCs, inner hair cells; OHCs, outer hair cells; IPC, inner pillar cells, DC, Deiters’ cells; IPhC, inner phalangeal cells. For the vestibule (right), insets are close-up views of the macula and ampulla cristae (scale bars **A**–**D**, 50 μm; insets, 5 μm). GFP-positive vestibular hair cells are outlined by dashed lines and cells other than sensory hair cells are indicated by arrowheads. (**E**) *Bar graphs* showing the percentage of hair cells—IHCs, OHCs and VHCs—transduced with AAV2 (black), AAV8 (pink), Anc80L65 (blue), and AAV9-PHP.eB (purple), (mean percentage ± SEM). Two-way ANOVA, **P < 0.01, ***P < 0.001 and ****P < 0.0001.

All four serotypes successfully transduced inner ear sensory cells, but the level of transduction rate varies depending on serotype, cell type and position along the cochlear spiral. AAV9-PHP.eB neonatal administration results in the highest transduction rate in cochlear sensory hair cells, with a mean rate of 85 ± 6% for IHCs and 85 ± 4.9% for OHCs (Fig. 2; Table 2). The IHC transduction efficiency was similar to that of Anc80L65, but significantly higher than that of AAV2 along the entire cochlear spiral (*p* = 0.0001, *p =* 0.0002, and *p =* 0.0007 for the apical, medial, and basal turns, respectively; two-way ANOVA) and it outperformed AAV8 in the basal part of the cochlea (Fig. 2, *p* = 0.0002, two-way ANOVA). Similarly, the rates of OHC transduction with AAV9-PHP.eB were markedly higher than those for AAV8 and AAV2 along the entire cochlear spiral (Fig. 2, *p* < 0.0001). By contrast to our observations at the embryonic stage, the transduction rate was much higher for IHCs than for OHCs for both AAV8 (59 ± 3.6% vs. 24 ± 4.6%, *p* = 0.0009, two-way ANOVA) and Anc80L65 (93 ± 4.1% vs. 60 ± 11.1%, *p* = 0.001, two-way ANOVA). The AAV2 serotype had the lowest IHC transduction efficiency.

Unlike injections during the embryonic stage, neonatal administrations resulted in a consistent transduction profile that followed an apico-basal gradient for both IHCs and OHCs, across all serotypes except AAV2 (Table 2). This gradient was particularly marked for AAV8, with IHC transduction rates of 90 ± 7.6%, 61 ± 9.0%, and 31 ± 12.1% for the apex, medial, and basal turns, respectively (*p* < 0.0001 for comparisons between the apex and base, *p* = 0.026 for comparisons between the apex and medial turn, and *p* = 0.023 for comparisons of the medial turn and base). Interestingly, SGNs and supporting cells, such as Deiters cells and inner pillar cells, were also targeted by AAV2 and Anc80L65, and to a lesser extent by AAV8 and AAV9-PHP.eB.

In the vestibule, the cellular transduction rates were similar in the ampulla cristae and the macula across all four serotypes (*ns*, two-way ANOVA, Table 2). The highest cellular transduction rate was achieved with Anc80L65 (82 ± 5.6%) while the lowest was obtained with AAV2 (14 ± 2.3%; *p* < 0.0001, one-way ANOVA, Fig. 2; Table 2).

**Table 2:** Transduction rates of inner ear hair cells after the injection of various AAV serotypes at the neonatal stage.

	AAV2	AAV8	Anc80L65	AAV9-PHP.eB	*p*
IHC	*n* = 4	*n* = 6	*n* = 5	*n* = 6	
Global	27 ± 6.1 [11–40]	59 ± 3.6 [41–71]	93 ± 4.1 [67–100]	85 ± 6 [60–100]	**0.0004**
Apex	26 ± 8.3 [1–38]	90 ± 7.6 [52–100]	94 ± 4.6 [63–100]	95 ± 5.0 [70–100]	**0.005**
Middle	26 ± 9.0 [1–40]	61 ± 9.0 [25–75]	91 ± 3.7 [80–100]	80 ± 10.0 [50–100]	**0.004**
Base	31 ± 5.2 [20–45]	31 ± 12.1 [6.3–85]	85 ± 10.6 [55–100]	81 ± 6.9 [60–100]	**0.005**
OHC	*n* = 5	*n* = 6	*n* = 4	*n* = 4	
Global	30 ± 9.8 [12–58]	24 ± 4.6 [5–35]	60 ± 11.1 [26–88]	85 ± 4.9 [71–97]	**0.007**
Apex	23 ± 13.1 [0–60]	29 ± 5.1 [8–49]	73 ± 7.9 [36–93]	96 ± 2.1 [88–100]	**0.0005**
Middle	24 ± 11.1 [10–57]	32 ± 11.4 [2–55]	51 ± 15.3 [14–82]	79 ± 7.5 [57–100]	**0.024**
Base	43 ± 10.2 [15–60]	18 ± 8.1 [0–46]	57 ± 14 [21–91]	80 ± 7.9 [60–96]	**0.0025**
VHC	*n* = 8	*n* = 8	*n* = 10	*n* = 18	
Global	14 ± 2.3 [1–25]	51 ± 5.5 [38–85]	82 ± 5.6 [50–98]	64 ± 3.9 [41–96]	**< 0.0001**
Ampulla	8 ± 2.9 [1–20]	55 ± 10.2 [40–85]	91 ± 2.1 [87–98]	55 ± 2.8 [45–70]	**0.0006**
Macula	18 ± 2.9 [9–25]	48 ± 5.2 [38–59]	74 ± 9.7 [50–90]	73 ± 6.1 [41–96]	**0.0043**

The data shown are the mean ± SEM [range]. Statistical comparisons were performed by two-way ANOVA. *N* is the number of organs analyzed. IHC = inner hair cells; OHC = outer hair cells; VHC = vestibular hair cells.

### Cell tropism of AAV in the mature inner ear

The injections were administered at different ages, covering key stages of cochlear maturation, specifically between P14 and P18 (early mature), shortly after hearing onset, and at P30 (late mature) once the auditory system is fully mature in mice. Inner-ear sensory epithelia were microdissected five days post-injection and immunolabeled as described above. For both stages, IHC transduction rates were robust along the entire cochlear spiral, while OHC transduction rates were lower, regardless of serotype (Tables 3 and 4). The transduction rate of cochlear sensory cells remained stable for AAV8 and Anc80L65 between P14-P18 and P30, however, declined for AAV9-PHP.eB and AAV2 (Tables 3 and 4).

**Fig. 3:**
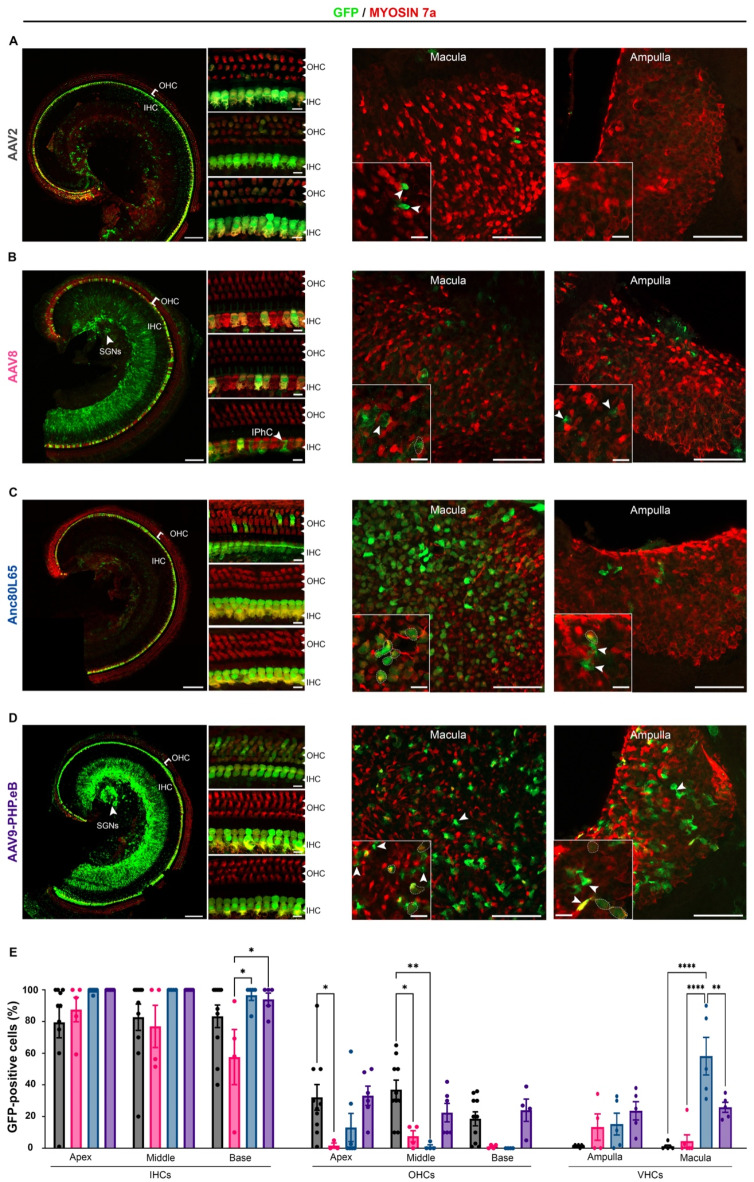
Comparison of the inner ear cell tropism of four AAV serotypes at early mature stage (P14-P18). Confocal images of the apical turn of the organ of Corti (left panels) and vestibular organs (right panels) after injection through the RWM at P14-18 with 2 µL of (**A**) AAV2-CBA-GFP, (**B**) AAV8-CMV-GFP, (**C**) AAV-Anc80L65-CMV-GFP or (**D**) AAV9-PHP.eB-CBA-GFP, followed by immunostaining, at P19-25, for GFP (green) and myosin 7a (red). For the cochlea (left), the insets provide higher-magnification views of the apical (top), medial (middle), and basal (bottom) regions (scale bars: **A**–**D**, 100 μm; insets, 5 μm). IHCs, inner hair cells; OHCs, outer hair cells; SGNs, spiral ganglion neurons. For the vestibule (right), broad view of the macula and ampulla cristae with corresponding close-up views (scale bars **A**–**D**, 50 μm; insets, 5 μm). GFP-positive vestibular hair cells are outlined by dashed lines and cells other than sensory hair cells are indicated by arrowheads. (**E**) *Bar graphs* showing the percentage of hair cells—IHCs, OHCs and VHCs—transduced with AAV2 (black), AAV8 (pink), Anc80L65 (blue), or AAV9-PHP.eB (purple), (mean percentage ± SEM). Two-way ANOVA, **P < 0.01, ***P < 0.001 and ****P < 0.0001.

With an IHC transduction rate of 99 ± 0.9% and 93 ± 2.8%, at P14-P18 and P30, respectively, Anc80L65 was the most efficient serotype for both stages (*p* = 0.009 and *p* = 0.0031 at P14-P18 and P30, respectively, two-way ANOVA). Concerning OHC, the highest transduction rates were obtained with AAV9-PHP.eB and AAV2 at the early mature stage, with a mean percentage of 26 ± 4.7% and 29 ± 5.5%, respectively, compared with AAV8 and Anc80L65 that transduced only 3 ± 1.5% and 7 ± 6.0%, respectively (*p* = 0.0004 and *p* = 0.006 for AAV8 compared with AAV2 and AAV9-PHP.eB, respectively; *p* = 0.0002 and *p* = 0.005 for Anc80L65 compared with AAV2 and AAV9-PHP.eB, respectively; two-way ANOVA) (Fig. 3; Table 3). However, at a later stage (P30), Anc80L65 and AAV2 showed relatively the highest OHC transduction efficiencies (13 ± 5.6% and 11 ± 3.4%, respectively), and AAV9-PHP.eB failed to infect OHCs (Table 4). For both stages and except for AAV2, the transduction rates of cochlear sensory hair cells were homogeneous across the entire cochlear spiral (ns, two-way ANOVA), in contrast to the embryonic and neonatal stages.

In vestibular organs, Anc80L65 gave the highest VHC transduction rates at both stages, infecting 37 ± 9.6% and 33 ± 5.2% of the VHCs at P14-P18 and P30, respectively (*p* = 0.0004 and *p* < 0.0001 respectively, two-way ANOVA, Tables 3 and 4). Interestingly, at both stages, the number of transduced hair cells with Anc80L65 was significantly higher in saccular and utricular VHCs compared to ampullar VHCs (*p* < 0.0001, two-way ANOVA, Tables 3 and 4). At P14-P18, AAV9-PHP.eB also transduced VHCs albeit to a lesser extent, with a transduction rate of 25 ± 3.1%, but the rates significantly dropped to 7 ± 3.1% when administered at P30.

**Fig. 4:**
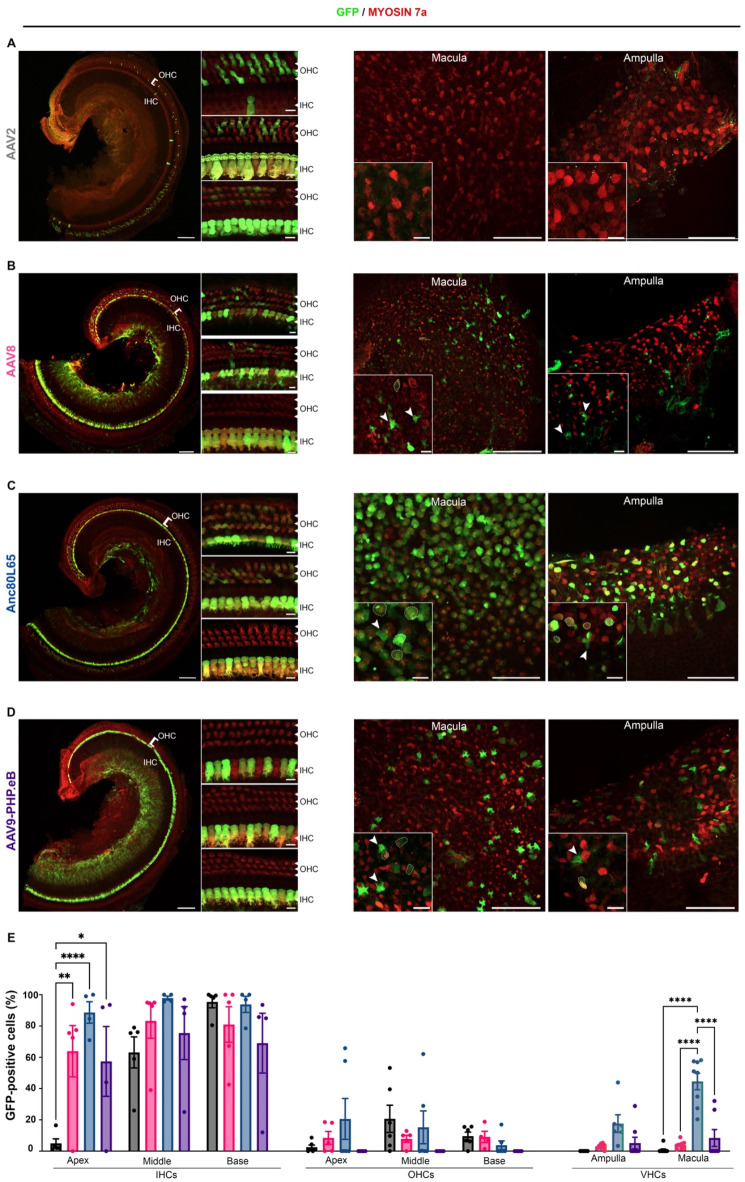
Comparison of the inner ear tropism of four AAV serotypes at late mature stage (P30). Confocal images of the apical turn of the organ of Corti (left panels) and vestibular organs (right panels) injected at P30 with 2 µl of (**A**) AAV2-CBA-GFP, (**B**) AAV8-CMV-GFP, (**C**) AAV-Anc80L65-CMV-GFP and (**D**) AAV9-PHP.eB-CBA-GFP via RWM, immunostained at P37 for GFP (green) and myosin 7a (red). For the cochlea, higher magnification insets of the apical (top), medial (middle), and basal (bottom) regions are inserted (scale bars: A-D, 100 μm; insets, 10 μm). On the right panel, large view of the macula and ampulla cristae with corresponding higher magnification (scale bars A-D, 50 μm; insets, 10 μm). (**E**) Comparative analyses of the transduction rate of inner hair cells (IHCs), outer hair cells (OHCs) and vestibular hair cells (VHCs) transduced with different serotypes including AAV2 (black), AAV8 (pink), Anc80L65 (blue), and AAV9-PHP.eB (purple), (mean percentage ± SEM). Two-way ANOVA, *P < 0.05, **P < 0.01, ***P < 0.001 and ****P < 0.0001.

The lowest transduction rates in VHC were observed with AAV8 (8 ± 5.4% and 3 ± 0.5, at P14-P18 and P30, respectively) and with AAV2, which infected almost no VHC at both stages (1 ± 0.4% and 0.5 ± 0.4% at P14-P18 and P30, respectively). Finally, for viral vectors that successfully transduced the vestibular end organs, cochlear delivery at the mature stage resulted in the transduction of vestibular supporting cells in addition to sensory hair cells (Figs. 3 and 4).

**Table 3:** Transduction rates of inner ear hair cells after the injection of various AAV serotypes at early mature stage (P14-P18).

	AAV2	AAV8	Anc80L65	AAV9-PHP.eB	*p*
IHC	*n* = 10	*n* = 4	*n* = 6	*n* = 6	
Global	82 ± 5.5 [53–100]	72 ± 8.2 [55–96]	99 ± 0.9 [94–100]	98 ± 1.1 [93–100]	**0.009**
Apex	79 ± 9.7 [1-100]	87 ± 7.6 [59–100]	99 ± 0.5 [96–100]	100 ± 0 [100–100]	**0.014**
Medial turn	83 ± 8.3 [20–100]	77 ± 13.3 [51–100]	100 ± 0 [100–100]	100 ± 0 [100–100]	0.085
Base	83 ± 7.1 [40–100]	58 ± 17.4 [10–93]	97 ± 3.3 [83–100]	94 ± 4.0 [80–100]	0.079
OHC	*n* = 10	*n* = 4	*n* = 6	*n* = 6	
Global	29 ± 5.5 [4–58]	3.4 ± 1.5 [0–7]	7 ± 6.0 [0–31]	26 ± 4.7 [10–39]	**0.007**
Apex	32 ± 8.1 [1–90]	2 ± 1.7 [0–5]	13 ± 8.9 [0–61]	33 ± 6.1 [10–50]	**0.026**
Medial turn	37 ± 6.0 [10–65]	8 ± 3.5 [0–14]	1 ± 1.1 [0–4]	22 ± 5.8 (10–42)	**0.004**
Base	19 ± 4.4 [1–36]	1 ± 0.6 [0–2]	0	24 ± 7.0 [5–39]	**0.003**
VHC	*n* = 7	*n* = 4	*n* = 5	*n* = 5	
Global	1 ± 0.4 [0–5]	8 ± 5.4 [0–34]	37 ± 9.6 [0–90]	25 ± 3.1 [6–38]	**0.0004**
Ampulla	1 ± 0.3 [0–2]	13 ± 8.3 [0–34]	15 ± 6.9 [0–32]	24 ± 5.8 [6–38]	**0.0004**
Macula	1 ± 0.8 [0–5]	4 ± 4 [0–24]	58 ± 11.8 [31–90]	26 ± 3.1 [18–35]	**0.003**

The data shown are the mean ± SEM [range]. Statistical comparisons were performed by two-way ANOVA. *N* is the number of organs analyzed. IHC = inner hair cells; OHC = outer hair cells; VHC = vestibular hair cells.

**Table 4:** Transduction rates of inner ear hair cells after injections of various AAV serotypes at late mature stage (P30).

	AAV2	AAV8	Anc80L65	AAV9-PHP.eB	*p*
IHC	*n* = 5	*n* = 5	*n* = 6	*n* = 5	
Global	55 ± 10 [1-100]	79 ± 6.3 [0-100]	93 ± 2.8 [71–100]	67 ± 10.5 [0–97]	**0.0031**
Apex	5 ± 2.9 [1–17]	64 ± 16.4 [0–94]	89 ± 6.8 [71–100]	57 ± 22.3 [0–94]	**0.005**
Medial turn	63 ± 9.9 [26–79]	83 ± 11.1 [39–95]	98 ± 1.2 [96–100]	75 ± 16.9 [25–97]	0.2
Base	95 ± 3.7 [80–100]	81 ± 11.4 [42–100]	94 ± 5.2 [79–100]	69 ± 19.1 [12–93]	0.3
OHC					
Global	11 ± 3.4 [0–53]	8 ± 1.8 [0–19]	13 ± 5.6 [0–66]	0	**0.046**
Apex	2.5 ± 1.4 [0–9]	8 ± 4.2 [0–19]	21 ± 13.1 [0–66]	0	0.2
Medial turn	21 ± 8.7 [0–53]	8 ± 2.2 [0–13]	15 ± 10.5 [0–62]	0	0.2
Base	10 ± 2.6 [0–16]	9 ± 3.6 [1–19]	4 ± 2.8 [0–17]	0	**0.035**
VHC					
Global	0.5 ± 0.4 [0–7]	3 ± 0.5 [0–9]	33 ± 5.2 [3–55]	7 ± 3.1 [0–32]	**< 0.0001**
Ampula	0	3 ± 0.6 [0–5]	18 ± 5.6 [3–40]	5 ± 3.7 [0–28]	**0.011**
Macula	1 ± 0.7 [0–7]	4 ± 0.9 [1–9]	44 ± 5.3 [20–58]	8 ± 5.4 [0–32]	**< 0.0001**

Data are mean ± SEM [range]. Statistical comparisons were performed using a two-way ANOVA. *N* is the number of organs analyzed. IHC = inner hair cells; OHC = outer hair cells; VHC = vestibular hair cells.

## Discussion

AAV-mediated gene therapy has given promising results for the treatment of hearing loss and balance disorders in a number of preclinical studies targeting various genetic forms of deafness^16^. In particular, recent clinical trials of AAV gene therapy for DFNB9 deafness have shown this approach to represent a significant breakthrough, opening up the possibility of curative treatments for other types of deafness of genetic origin, with or without associated vestibular defects^11,12^. The cell tropism and transduction efficiency of AAV vectors within the inner ear are key factors influencing the outcome of gene therapy. The human inner ear matures much earlier during development than that of the mouse. The identification of highly specific AAV serotypes that target inner ear cells efficiently at given developmental stages is therefore a crucial step not only for basic research but also for laying the groundwork for preclinical studies. Evaluation of the specific tropism of AAVs for VHCs is also crucial for the effective restoration of vestibular function to ensure that gene therapy can address both auditory and balance functions. These aspects are crucial for ensuring successful outcomes and reaching translational goals.

We aimed to bridge this gap by comprehensively investigating the tropism for inner ear hair cells of various AAV serotypes in mice at different developmental stages. Four AAV serotypes known for their efficiency in targeting sensory hair cells — AAV2, AAV8, Anc80L65, and AAV9-PHP.eB — were injected into the inner ears of mice at the embryonic, neonatal, and mature stages. The rates of inner-ear hair cell transduction were highly variable when these four serotypes were administered at embryonic stage E14, but a consistent pattern emerged in the cochlear spiral and in the vestibular end organs. The transduction rates of both IHCs and OHCs followed a baso-apical gradient, with hair cells located at the base being more efficiently transduced than those at the apex of the cochlea. Furthermore, at this embryonic stage OHCs were typically more efficiently transduced than IHCs. Previous studies analyzing the outcomes of embryonic injections with similar serotypes (AAV8 and Anc80L65) showed no baso-apical gradient and a robust transduction of both OHCs, IHCs, and supporting cells^17,18^. This discrepancy is likely due to the fact that administration was performed at E12 stage, when the cells are predominantly bipotent, rendering them more naive and permissive to AAV transduction^19^. Interestingly, when administered at the neonatal stage (P2), AAV8, Anc80L65, and AAV9-PHP.eB resulted in higher rates of hair cell transduction than AAV2, consistent with previous studies^20,21^. Furthermore, hair cell transduction rates followed an apical-to-basal gradient opposite to that observed following injection into the cochlea at E14. It is noteworthy that the OHCs become less permissive to AAV transduction when these serotypes are injected at a later stage, as evidenced by a significant decrease in their transduction rate compared to IHCs, which is nearly reaching zero (e.g. AAV9-PHP.eB) at the mature stage.

Interestingly, the decrease in basal-to-apical gradients of hair cell transduction rate following AAV administration at the neonatal stage mirrored the basal-to-apical wave of differentiation/maturation observed in cochlear hair cells. This pattern of hair cell differentiation/maturation is tightly regulated by interactions between ligands and their receptors, involving heparan sulfate proteoglycans (HSPGs)^22^. A temporal wave of HSPG expression along the apical-to-basal axis of the cochlea has been described^23^. HSPGs are initially expressed in hair cells at the apex and, over time, their expression is gradually activated in basal hair cells and downregulated in apical hair cells. As HSPGs also function as the primary attachment receptors for AAVs, variations in their concentration and composition during the baso-to-apical wave of differentiation and maturation may affect the transduction efficiency of AAVs, accounting for the observed variability.

This statement is consistent with the finding that the basal-to-apical distribution profile of transduced hair cells almost entirely disappears when these AAVs are injected into the mature cochlea. However, OHCs were consistently more resistant to transduction with all the serotypes tested in both this and previous studies^24,25^.

One of the most striking observations concerns AAV9-PHP.eB, which transduced the smallest numbers of cochlear sensory hair cells at the embryonic stage but a significant number of neurons, as demonstrated by the presence of varicosities highly immunoreactive to GFP antibody at the base of the IHCs, corresponding to the neurons in the process of establishing connections with these cells. By contrast, administration at the neonatal and mature stages resulted in the highest rates of transduction for both IHCs and OHCs, with hair cell transduction rates following an apical-to-basal gradient at the neonatal stage. The tissue tropism of this serotype is determined principally by its attachment receptor, notably the N-linked galactose of the heparan sulfate proteoglycan, and its coreceptor, the 37/67-kDa laminin^26^. Transduction efficiency extended to hair cells at the neonatal and mature stages, suggesting that these two components are probably well represented on the surface of hair cells during these stages. These findings suggest that AAV9-PHP.eB could potentially be used for the in vivo genetic manipulation of cochlear neurons at the embryonic stage and for targeting both hair cells and auditory neurons at the postnatal and mature stages.

Similarly, AAV2 transduced relatively small numbers of IHCs and OHCs at the embryonic and neonatal stages. However, at the adult stage, it transduced predominantly IHCs, for which the transduction rate was almost 100% rate. AAV2 has been shown to interact functionally predominantly with the second Ig-like polycystic kidney disease (PKD) repeat domain (PKD2)^27^. Our findings suggest that, in the mature cochlea, corresponding to a therapeutic time window transposable to clinical practice in humans, the surface glycan composition of IHCs is optimal for AAV2 attachment and transduction whereas that of OHCs is not. Similar findings have been reported for several AAV serotypes tested for use in gene therapy for deafness^28–32^. The recent breakthrough obtained for DFNB9 gene therapy was possible only because the otoferlin protein is expressed by IHCs, the cells preferentially targeted by AAVs at this stage^33^. Unfortunately, the vast majority of genetic causes of hearing loss involve both IHCs and OHCs^34^. The identification of potential AAV-binding receptors common to both IHCs and OHCs at the mature stage will, therefore, undoubtedly improve vector design and the selection of appropriate serotypes, in turn improving the outcomes of AAV gene therapy for deafness involving these cells.

In the vestibular organs, we observed similar almost homogeneous levels of VHC transduction with AAV8, Anc80L65, and AAV9-PHP.eB at the embryonic and neonatal stages, consistent with published findings^18,20,30^. However, transduction rates were much lower at the mature stage, particularly for AAV8. Nevertheless, for Anc80L65 — the serotype for which VHC transduction rates were highest at mature stages — transduction rates were much higher in the utricular and saccular maculae than in the crista ampullae. Accordingly, in a Usher syndrome mouse model, the rescue of otolithic function was significantly improved compared to that of the canal function after viral gene therapy using an Anc80L65 serotype at a mature stage^35^. This difference in transduction rates between otolithic organs and semicircular canals may reflect poor diffusion of the viral vector to the semicircular canals, which are farther from the injection site when injections are performed through the RWM.

In conclusion, our findings demonstrate the age-dependent efficiency of inner ear sensory hair cell targeting by AAV serotypes, with a tropism for cochlear and vestibular hair cells that varies considerably, depending on the state of maturation of the inner ear. We focused here on the study of inner ear hair cells, which are the primary site of abnormalities in the majority of non-syndromic genetic deafness^4,5^. However, as the most frequent congenital deafness is related to *GJB2*, which is expressed in non-sensory epithelial cells and lateral wall^35^, it would be interesting to conduct a similar study using vectors specifically targeting these cells, such as AAV-DJ^36^, AAV8BP2^37^ or AAV1^38^. Identification of the optimal serotype for a particular type of target cell is essential, particularly for interventions at the mature stage, and may require the development of engineered AAV through directed evolution, the identification of AAV receptors within the inner ear, or the use of a hair cell-specific promoter. Such approaches could potentially improve targeting efficiency for sensory hair cells, paving the way for new translational opportunities for human gene therapy.

## Methods

### Mice

All experiments were carried out in accordance with relevant guidelines and regulations and approved by the Institute Pasteur ethics committee and the *Institut National de la Santé et de la Recherche Médicale* (INSERM). Wild-type C57/Bl6 mice (both male and female) from Janvier Labs (France) were used at the various stages and were randomly assigned to the different experimental groups. All animals were housed at the animal facility of the *Institut de l’Audition* which has French Ministry of Agriculture accreditation.

### Viral vectors

All the viral constructs expressed the GFP gene under the control of ubiquitous promoters, such as the cytomegalovirus (CMV) and chicken beta actin (CBA) promoters. The vectors were produced at different viral manufacturing facilities and the preparations used had titers between 1 and 2 × 10^13^ vg/ml, depending on the production site. AAV2-CBA-GFP was obtained from Penn Vector Core at a titer of 1.04 × 10^13^ vg/ml, AAV9-PHP.eB-CBA-GFP was obtained from the viral vector facility of Zurich University at a titer of 2 × 10^13^ vg/ml, AAV8-CMV-GFP was obtained from Addgene at a titer of 2.3 × 10^13^ vg/ml and Anc80L65-CMV-GFP was produced by SignaGen Laboratories at titer of 1.05 × 10^13^ vg/ml. Aliquots of all the vectors were stored at -80 °C until use.

### Surgery

Mice were anesthetized with isoflurane via a face mask suitable for the age of the mouse concerned (induction at 4% then maintenance at 2%). Analgesia was achieved with local anesthesia (5 mg/kg lidocaine at the surgical site) and 0.2 mg/kg meloxicam (Metacam^®^) for adult mice. The mice were placed on a heating pad during the procedure to maintain body temperature at 37 °C. The eyes of adult mice were protected with Ocrygel^®^.

The intracochlear injections were performed as previously described^30,35,36^. For postnatal injections, a retro-auricular incision was made on the left ear and the cervical muscles were gently dissected. At the neonatal stage, the round window was identified beneath the facial nerve whereas, at the mature stage, the otic bulla was exposed and opened to access the round window niche. At both stages, the RWM was punctured with a glass pipette, leading to the leakage of perilymphatic fluid. The injection was performed with a pulled glass pipette (obtained with a Sutter^®^ P-97 micropipette puller). We injected 2 µL of viral preparation at a speed of 100 µL/s with an automatic microinjector (WPI^®^ Injector Nanoliter 2020). The RWM was then sealed with a graft of muscle and fat and the incision was closed with Vetbond.

For injections into embryos, pregnant mice (between days 13 and 15 of gestation - E13 to E15) were placed in a supine position on a heating pad. A 1.5 cm vertical midline incision was made into the skin of the anterior abdominal wall. An incision was then made into the peritoneum and the uterine horns were gently externalized and placed on sterile compresses. The uterine horns and abdominal cavity were regularly irrigated with saline heated at 37°C. Injection into the otocyst of the embryo was performed with a glass pipette (Sutter^®^ P-97 micropipette puller: pressure 200, temperature + 3 units, pull = 0, speed = 46, time = 110) under a binocular magnifying glass. The relief of the ear was identified in the center of a square traced by the main cephalic vein and its anterior and posterior branches^37^ and 1 µL of virus preparation was injected into one of the two ears with a nanoliter injector (WPI^®^, Sarasota, USA) at a speed of 100 nL/s. The uterus was replaced in the abdominal cavity once the injections into the embryos had been performed and the abdominal incisions were closed with absorbable sutures (Vicryl^®^ 5/0).

### Immunochemistry and counting

Mice were euthanized by decapitation before P10 and by cervical dislocation under general anesthesia (ketamine - Imalgene^®^, 100 mg/kg and xylazine chlorydrate - Rompun^®^ 2%, 10 mg/kg diluted in PBS) after P20. After dissection, the inner ears were harvested and fixed by incubation with 4% paraformaldehyde in PBS for 45 min at room temperature. For mice undergoing injections at the mature stage, an additional decalcification step involving overnight incubation with ethylenediamine tetra-acetic acid (EDTA; 0.5 M, pH 7.4) was performed at 4 °C. The cochleae and vestibular organs were then rinsed three times in PBS for 10 min each, microdissected, and incubated for 1 h at room temperature in blocking solution (PBS supplemented with 20% normal horse serum and 0.3% Triton X-100).

The samples were then incubated overnight with the primary antibodies: chicken anti-GFP (1:500; Abcam) and rabbit anti-myosin VI (1:500, Proteus Biosciences) antibodies in PBS supplemented with 5% of the blocking solution. The samples were rinsed three times for 10 min each in PBS and then incubated for 1 h with the secondary antibodies: ATTO-488–conjugated goat anti-chicken IgG antibody (1:500 dilution; Sigma–Aldrich) and ATTO-647-conjugated goat anti-rabbit IgG antibody (1:500 dilution; Sigma–Aldrich). Samples were then mounted in Fluorsave (Calbiochem, USA) and z-stack images were captured with a Zeiss LSM-900 confocal microscope equipped with a Plan Apochromat 63×/1.4 N.A. oil immersion lens (Carl Zeiss).

Images were analyzed with ImageJ2 (version 2.9.0/1.53T), and the transduction rate was determined as the percentage of GFP-positive cells among hair cells labeled with the anti-myosin VI antibody. For vestibular organs, the analysis comprises all VHCs, without differentiation between type 1 and type 2 hair cells.

### Statistics

All data are reported in accordance with ARRIVE guidelines. All the cell-count data are presented as the mean percentage ± SEM and the statistical analysis was performed with PRISM 9.5.1 software (GraphPad, San Diego, CA, USA). We used one-way or two-way ANOVA to compare two or more experimental groups for data following a normal distribution, and Kruskal-Wallis tests if the data were non-normal. Tukey’s post hoc test was used to correct for multiple comparisons, and *p*-values below 0.01 were considered significant.

## Data Availability

The data supporting the findings of this study are available within the article.
